# The genome sequence of a hoverfly *Eristalinus aeneus *(Scopoli, 1763)

**DOI:** 10.12688/wellcomeopenres.20636.2

**Published:** 2024-05-24

**Authors:** Olga Sivell, Chris Raper, Ryan Mitchell, Duncan Sivell, Natural History Museum Genome Acquisition Lab, Darwin Tree of Life Barcoding collective, Wellcome Sanger Institute Tree of Life Management, Samples and Laboratory team, Wellcome Sanger Institute Scientific Operations: Sequencing Operations, Wellcome Sanger Institute Tree of Life Core Informatics team, Tree of Life Core Informatics collective, Darwin Tree of Life Consortium

**Affiliations:** 1 Natural History Museum, London, England, UK; 2 Independent researcher, Sligo Town, County Sligo, Ireland

**Keywords:** Eristalinus aeneus, hoverfly, genome sequence, chromosomal, Diptera

## Abstract

We present a genome assembly from an individual female *Eristalinus aeneus* (a hoverfly; Arthropoda; Insecta; Diptera; Syrphidae). The genome sequence is 495.4 megabases in span. Most of the assembly is scaffolded into 6 chromosomal pseudomolecules. The mitochondrial genome has also been assembled and is 15.97 kilobases in length.

## Species taxonomy

Eukaryota; Metazoa; Eumetazoa; Bilateria; Protostomia; Ecdysozoa; Panarthropoda; Arthropoda; Mandibulata; Pancrustacea; Hexapoda; Insecta; Dicondylia; Pterygota; Neoptera; Endopterygota; Diptera; Brachycera; Muscomorpha; Eremoneura; Cyclorrhapha; Aschiza; Syrphoidea; Syrphidae; Eristalinae; Eristalini; *Eristalinus*; *Eristalinus aeneus* (Scopoli, 1763) (NCBI:txid2017640).

## Background

The genus *Eristalinus* is represented in Europe by four species, only two of which: *E. aeneus* (Scopoli, 1763) and *E. sepulchralis* (Linnaeus, 1758) occur in Britain ( Ball & Morris, 2015; Chandler, 2023; Speight, 2017). Both *E. sepulchralis* and *E. aeneus* are dark, shiny and slightly bronze in appearance. They both have spotted eyes and a loop in the wing vein R _4+5_ that distinguishes them from other British hoverflies. They can be separated based on the extent of hairs on the eye: in *E. sepulchralis* the whole eye is hairy while *E. aeneus* does not have hairs on the lower part of the eye ( Ball & Morris, 2015; Falk *et al.*, 2023; Stubbs & Falk, 2002; van Veen, 2014). In Europe the females of these species can be separated by the appearance of the frons and vertex: in *E. aeneus* the area around the ocellar triangle and often also along the upper margins of the compound eyes is dark and lustrous, while in *E. sepulchralis* it is dull or slightly shiny and uniformly grey dusted ( Kahanpää, 2022). The males can be identified by the width of the gap between the eyes: these are clearly separated in *E. sepulchralis* but holoptic in *E. aeneus* ( Kahanpää, 2022; van Veen, 2014). Male genitalia are distinct ( Pérez-Banón *et al.*, 2003).

*Eristalinus aeneus* is a widespread and cosmopolitan species with a Holarctic, Oriental, Afrotropical and Australasian distribution that includes Hawaii, Mauritius, Bermuda and the Gilbert and Ellis Islands. In the Afrotropics it extends south to Tanzania and in the Nearctic it reaches California and Texas ( Speight, 2017).

Through most of its range *E. aeneus* can be found near ponds, slow-moving rivers, streams, irrigation ditches and coastal lagoons. In southern Europe the larvae of *E. aeneus* were found in association with animal dung and sewage farms ( Speight, 2017).

In the northern limits of its range, including Britain and Ireland, this species is almost exclusively coastal where the larvae live in rotting seaweed in brackish waters or in rock pools ( Ball & Morris, 2015; Stubbs & Falk, 2002). According to Hartley (1961), there are two generations per year. *Eristalinus aeneus* is widely distributed around the British coastline but is more frequently recorded in the south ( Ball *et al.*, 2011). The flight period is generally from March to November, peaking in July and August, although adults can be encountered in any month of the year ( Ball *et al.*, 2011). This hoverfly overwinters as an adult and has been found hibernating in buildings ( Hartley, 1961).

The adults visit a variety of flowers, mainly yellow composites, white umbellifers, Aster, hoary alison *Berteroa incana*, rock-rose *Cistus*, oregano *Origanum*, creeping willow *Salix repens*, ragworts and groundsels *Senecio*, and dandelions *Taraxacum* ( Speight, 2017) as well as other plants, such as *Thymelaea velutina*, endemic to the Balearic Islands ( De La Bandera & Traveset, 2006). This species was also found to be an effective pollinator of various crops, for example mango *Mangifera indica*, watermelon *Citrullus lanatus*, onion *Allium cepa*, chickpea *Cicer arietinum*, celery *Apium graveolens* and fennel *Foeniculum vulgare* ( Latif *et al.*, 2019; Saeed *et al.*, 2008; Sánchez *et al.*, 2022a; Sánchez *et al.*, 2022b; Sánchez *et al.*, 2022c).

The third instar larva and puparium of *E. aeneus* were described by Hartley (1961). Pérez-Bañón *et al*. (2003) also published a description of the third instar larva and puparium with SEM images of the anterior larval and pupal spiracles and a key to the puparia of European *Eristalinus* species. The intra-puparial development of *Eristalinus aeneus* was researched by Campoy *et al*. (2020).

Pérez-Bañón *et al.* (2003) also published molecular data (mitochondrial COI and nuclear 28S rDNA) for all the European species and *E. dubiosus* (Curran, 1939) from Kenya, as well as a few other species from the tribe Eristalini. The proposed phylogeny based on their findings (molecular and male genitalia) divides species into two clades (genera/subgenera), placing *E. aeneus* together with *E. sepulchralis* in *Eristalinus* Rondani, 1845, while the Mediterranean species *E. taeniops* (Wiedemann, 1818) and *E. megacephalus* (Rossi, 1794) belong in *Eristalodes* Mik, 1897. However, further studies are needed to resolve their taxonomic status ( Pérez-Banón *et al.*, 2003). 

Here we present a high-quality genome of *E. aeneus.* It was sequenced based on one female specimen from Mullion, The Lizard National Nature Reserve, Cornwall, England. The genome of the other British *Eristalinus* species *, E. sepulchralis*, was published by Falk *et al.* (2023). Both these genomes will aid research on the taxonomy and phylogeny of *Eristalinus* and related species. The genomes have been generated as part of the Darwin Tree of Life Project, a collaborative effort to sequence all named eukaryotic species in the Atlantic Archipelago of Britain and Ireland.

## Genome sequence report

**Figure 1.**
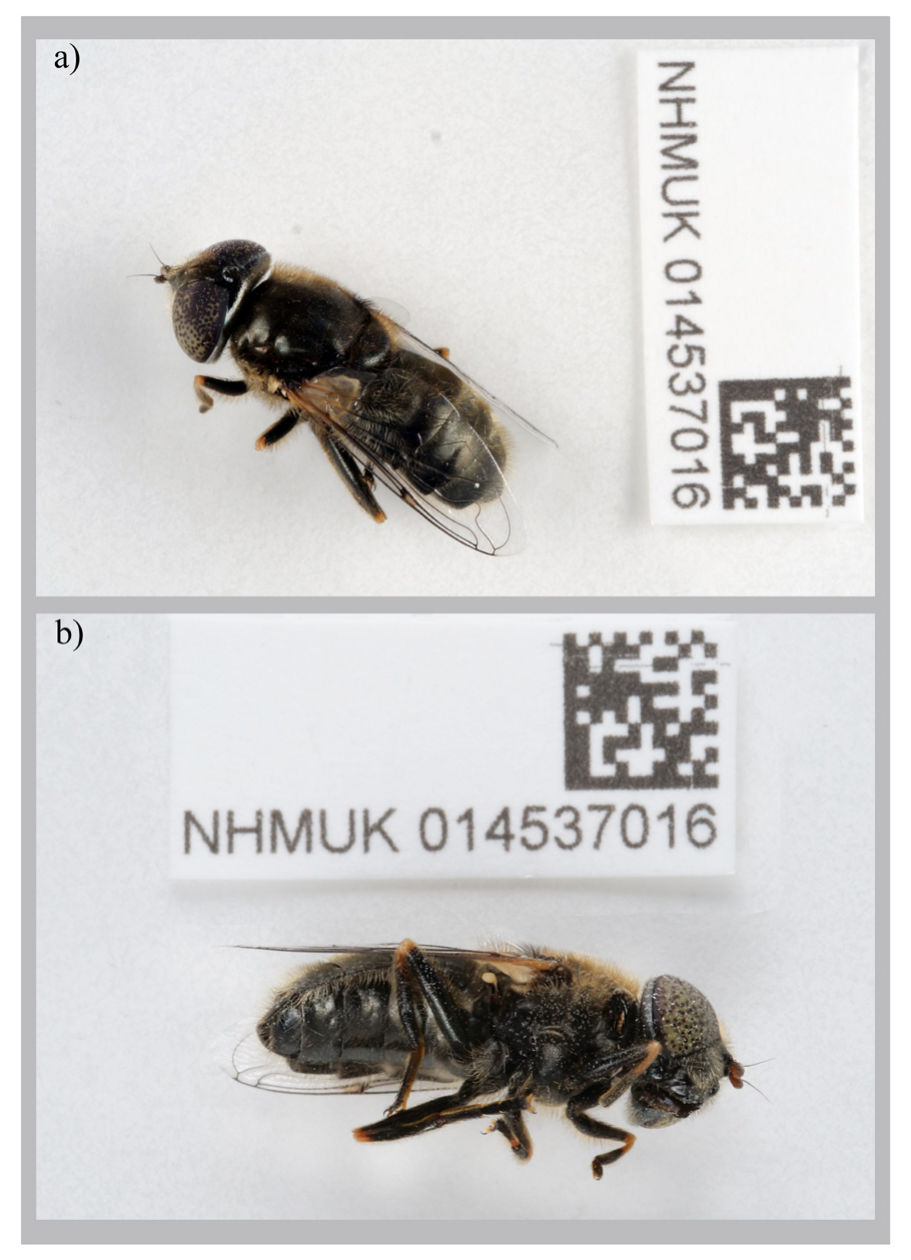
Photographs of the *Eristalinus aeneus* (idEriAene1, NHMUK014537016) specimen used for genome sequencing **a**) dorsal view, **b**) latero-ventral view.

The genome was sequenced from a female *Eristalinus aeneus* ( Figure 1) collected from Mullion, England (50.02, –5.24). A total of 56-fold coverage in Pacific Biosciences single-molecule HiFi long reads was generated. Primary assembly contigs were scaffolded with chromosome conformation Hi-C data. Manual assembly curation corrected 85 missing joins or mis-joins and removed one haplotypic duplication, reducing the scaffold number by 17.15%, and increasing the scaffold N50 by 9.65%.

**Figure 2.**
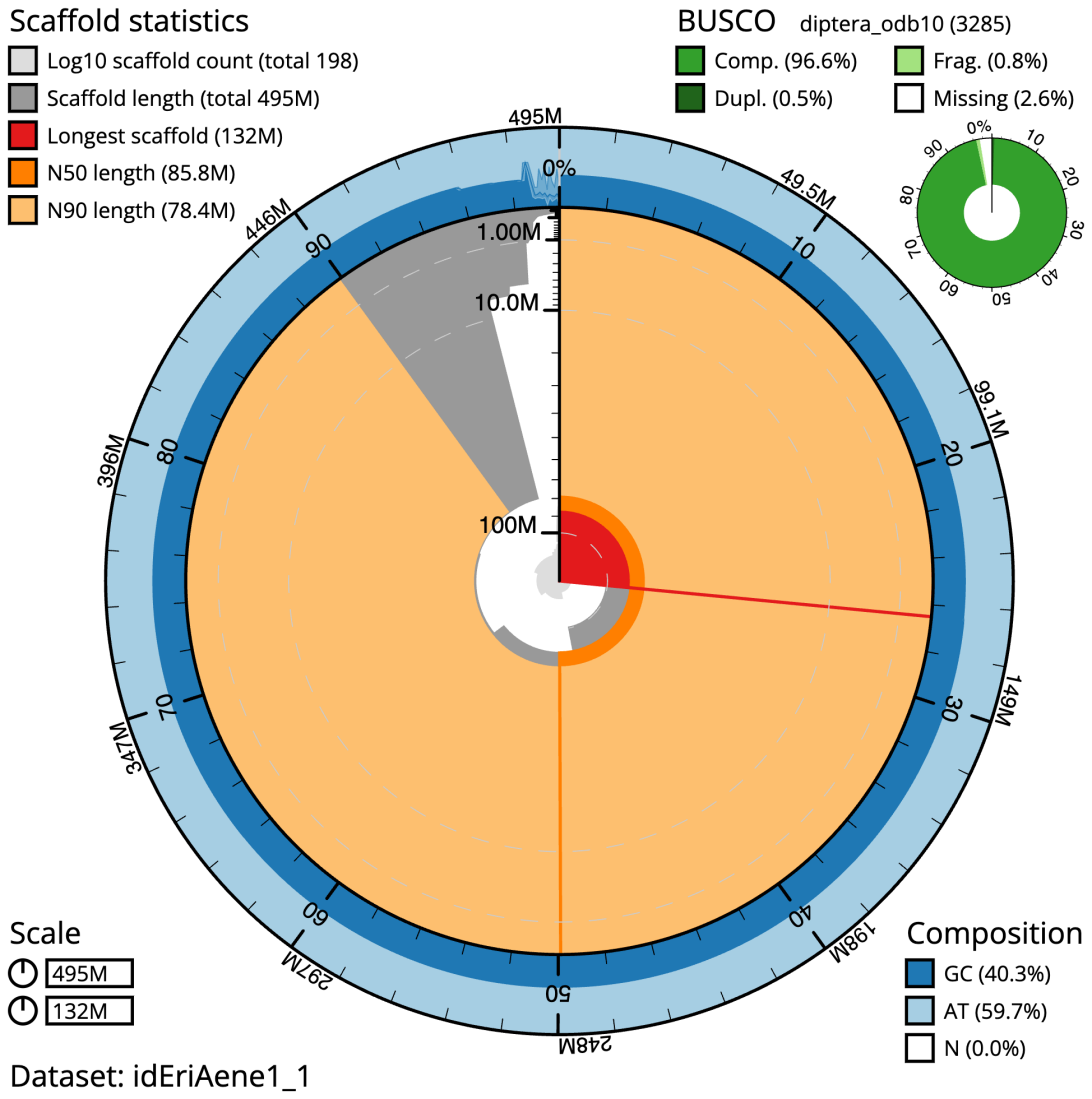
Genome assembly of *Eristalinus aeneus*, idEriAene1.1: metrics. The BlobToolKit snailplot shows N50 metrics and BUSCO gene completeness. The main plot is divided into 1,000 size-ordered bins around the circumference with each bin representing 0.1% of the 495,389,070 bp assembly. The distribution of scaffold lengths is shown in dark grey with the plot radius scaled to the longest scaffold present in the assembly (131,691,179 bp, shown in red). Orange and pale-orange arcs show the N50 and N90 scaffold lengths (85,846,282 and 78,373,126 bp), respectively. The pale grey spiral shows the cumulative scaffold count on a log scale with white scale lines showing successive orders of magnitude. The blue and pale-blue area around the outside of the plot shows the distribution of GC, AT and N percentages in the same bins as the inner plot. A summary of complete, fragmented, duplicated and missing BUSCO genes in the diptera_odb10 set is shown in the top right. An interactive version of this figure is available at https://blobtoolkit.genomehubs.org/view/idEriAene1_1/dataset/idEriAene1_1/snail.

**Figure 3.**
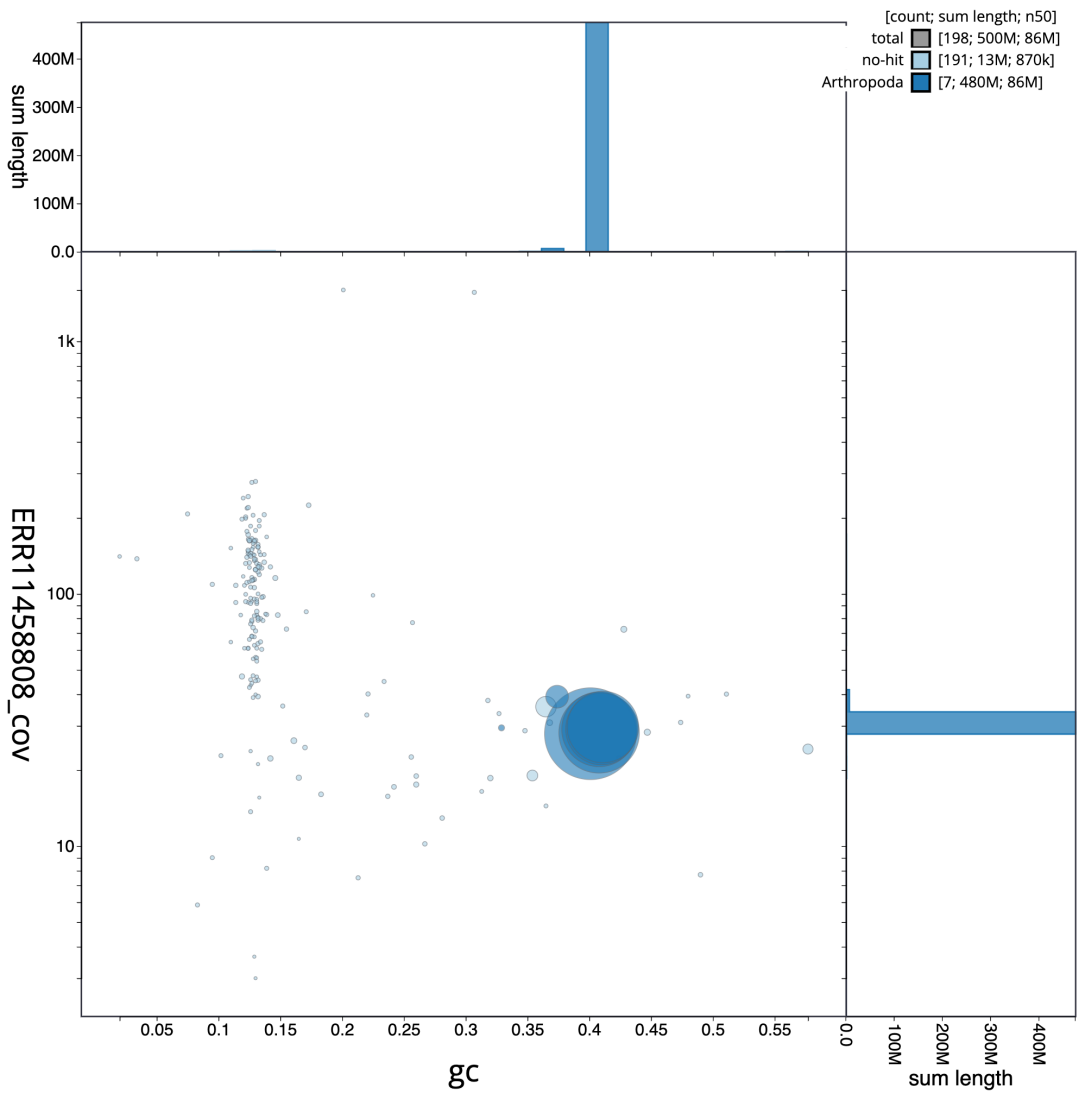
Genome assembly of *Eristalinus aeneus*, idEriAene1.1: BlobToolKit GC-coverage plot. Scaffolds are coloured by phylum. Circles are sized in proportion to scaffold length. Histograms show the distribution of scaffold length sum along each axis. An interactive version of this figure is available at https://blobtoolkit.genomehubs.org/view/idEriAene1_1/dataset/idEriAene1_1/blob.

**Figure 4.**
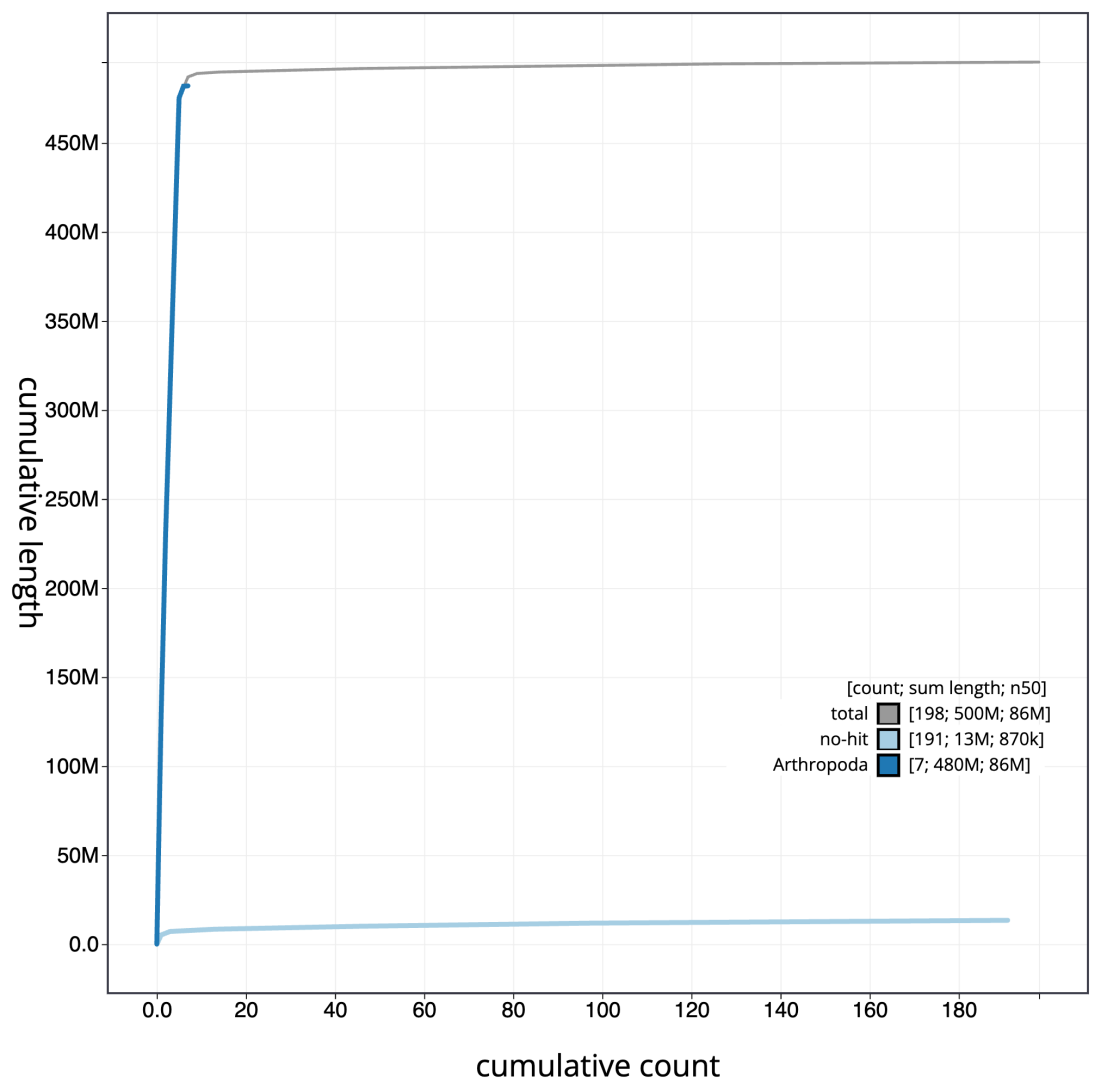
Genome assembly of *Eristalinus aeneus*, idEriAene1.1: BlobToolKit cumulative sequence plot. The grey line shows cumulative length for all scaffolds. Coloured lines show cumulative lengths of scaffolds assigned to each phylum using the buscogenes taxrule. An interactive version of this figure is available at https://blobtoolkit.genomehubs.org/view/idEriAene1_1/dataset/idEriAene1_1/cumulative.

**Figure 5.**
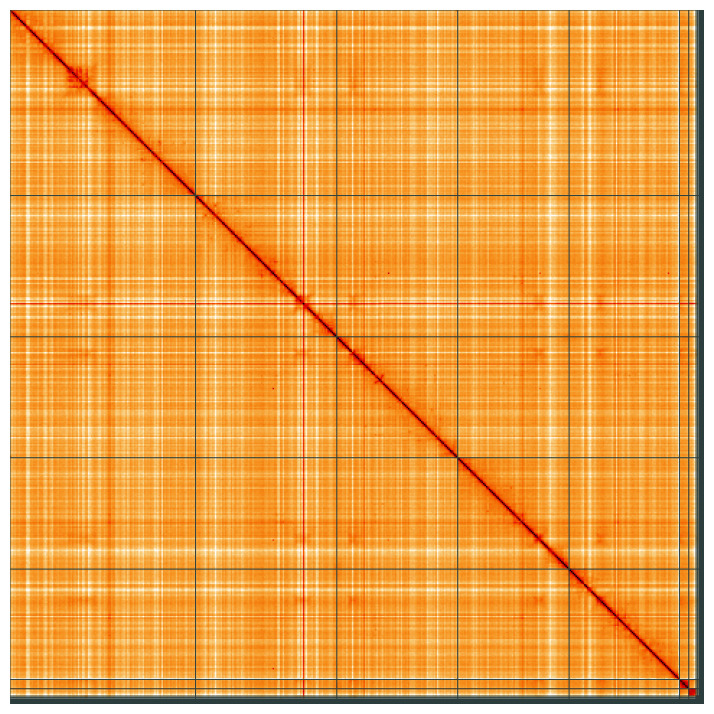
Genome assembly of *Eristalinus aeneus*, idEriAene1.1: Hi-C contact map of the idEriAene1.1 assembly, visualised using HiGlass. Chromosomes are shown in order of size from left to right and top to bottom. An interactive version of this figure may be viewed at https://genome-note-higlass.tol.sanger.ac.uk/l/?d=TFGREwXKStWHgFtrW8UVWQ.

The final assembly has a total length of 495.4 Mb in 198 sequence scaffolds with a scaffold N50 of 85.8 Mb ( Table 1). The snail plot in Figure 2 provides a summary of the assembly statistics, while the distribution of assembly scaffolds on GC proportion and coverage is shown in Figure 3. The cumulative assembly plot in Figure 4 shows curves for subsets of scaffolds assigned to different phyla. Most (97.3%) of the assembly sequence was assigned to 6 chromosomal-level scaffolds, representing 6 autosomes. Chromosome-scale scaffolds confirmed by the Hi-C data are named in order of size ( Figure 5; Table 2). While not fully phased, the assembly deposited is of one haplotype. Contigs corresponding to the second haplotype have also been deposited. The mitochondrial genome was also assembled and can be found as a contig within the multifasta file of the genome submission.

**Table 1.** Genome data for *Eristalinus aeneus*, idEriAene1.1.

Project accession data		
Assembly identifier	idEriAene1.1	
Species	*Eristalinus aeneus*	
Specimen	idEriAene1	
NCBI taxonomy ID	2017640	
BioProject	PRJEB62161	
BioSample ID	SAMEA112221990	
Isolate information	idEriAene1, female: whole organism (DNA sequencing and Hi-C sequencing)	
Assembly metrics *		*Benchmark*
Consensus quality (QV)	61.2	*≥ 50*
*k*-mer completeness	100.0%	*≥ 95%*
BUSCO **	C:96.6%[S:96.1%,D:0.5%],F:0.8%,M:2.6%,n:3,285	*C ≥ 95%*
Percentage of assembly mapped to chromosomes	97.3%	*≥ 95%*
Sex chromosomes	Not identified	*localised homologous pairs*
Organelles	Mitochondrial genome: 15.97 kb	*complete single alleles*
Raw data accessions		
PacificBiosciences SEQUEL II	ERR11458808	
Hi-C Illumina	ERR11468733	
Genome assembly		
Assembly accession	GCA_955652365.1	
*Accession of alternate haplotype*	GCA_955652355.1	
Span (Mb)	495.4	
Number of contigs	448	
Contig N50 length (Mb)	5.3	
Number of scaffolds	198	
Scaffold N50 length (Mb)	85.8	
Longest scaffold (Mb)	131.69	

* Assembly metric benchmarks are adapted from column VGP-2020 of “Table 1: Proposed standards and metrics for defining genome assembly quality” from ( Rhie *et al.*, 2021). ** BUSCO scores based on the diptera_odb10 BUSCO set using version 5.3.2. C = complete [S = single copy, D = duplicated], F = fragmented, M = missing, n = number of orthologues in comparison. A full set of BUSCO scores is available at https://blobtoolkit.genomehubs.org/view/idEriAene1_1/dataset/idEriAene1_1/busco.

**Table 2.** Chromosomal pseudomolecules in the genome assembly of *Eristalinus aeneus*, idEriAene1.

INSDC accession	Chromosome	Length (Mb)	GC%
OY019130.1	1	131.69	40.0
OY019131.1	2	100.31	41.0
OY019132.1	3	85.85	41.0
OY019133.1	4	79.05	41.0
OY019134.1	5	78.37	41.0
OY019135.1	6	6.56	37.5
OY019136.1	MT	0.02	20.0

The estimated Quality Value (QV) of the final assembly is 61.2 with *k*-mer completeness of 100.0%, and the assembly has a BUSCO v5.3.2 completeness of 96.6% (single = 96.1%, duplicated = 0.5%), using the diptera_odb10 reference set ( *n* = 3,285).

Metadata for specimens, barcode results, spectra estimates, sequencing runs, contaminants and pre-curation assembly statistics are given at https://links.tol.sanger.ac.uk/species/2017640.

## Methods

### Sample acquisition and nucleic acid extraction

A female *Eristalinus aeneus* (specimen ID NHMUK014537016, ToLID idEriAene1) was collected from Mullion, Cornwall, England. The Lizard National Nature Reserve, England, UK (latitude 50.02, longitude –5.24) on 2021-07-01 using an aerial net. The specimen was collected by Olga Sivell and Chris Raper (Natural History Museum) and identified by Ryan Mitchell (Oxford University Museum of Natural History) and dry frozen at –80 °C.

The workflow for high molecular weight (HMW) DNA extraction at the Wellcome Sanger Institute (WSI) includes a sequence of core procedures: sample preparation; sample homogenisation, DNA extraction, fragmentation, and clean-up. For sample preparation, the idEriAene1 sample was weighed and dissected on dry ice ( Jay *et al.*, 2023). Tissue from the whole organism was homogenised using a PowerMasher II tissue disruptor ( Denton *et al.*, 2023a). HMW DNA was extracted using the Automated MagAttract v2 protocol ( Oatley *et al.*, 2023a). The DNA was sheared into an average fragment size of 12–20 kb in a Megaruptor 3 system with speed setting 31 ( Bates *et al.*, 2023). Sheared DNA was purified by solid-phase reversible immobilisation ( Oatley *et al.*, 2023b): in brief, the method employs a 1.8X ratio of AMPure PB beads to sample to eliminate shorter fragments and concentrate the DNA. The concentration of the sheared and purified DNA was assessed using a Nanodrop spectrophotometer and Qubit Fluorometer and Qubit dsDNA High Sensitivity Assay kit. Fragment size distribution was evaluated by running the sample on the FemtoPulse system.

Protocols developed by the WSI Tree of Life core laboratory have been deposited on protocols.io ( Denton *et al.*, 2023b).

### Sequencing

Pacific Biosciences HiFi circular consensus DNA sequencing libraries were constructed according to the manufacturers’ instructions. DNA sequencing was performed by the Scientific Operations core at the WSI on a Pacific Biosciences SEQUEL II instrument. Hi-C data were also generated from remaining tissue of idEriAene1 using the Arima2 kit and sequenced on the Illumina NovaSeq 6000 instrument.

### Genome assembly, curation and evaluation

Assembly was carried out with Hifiasm ( Cheng *et al.*, 2021) and haplotypic duplication was identified and removed with purge_dups ( Guan *et al.*, 2020). The assembly was then scaffolded with Hi-C data ( Rao *et al.*, 2014) using YaHS ( Zhou *et al.*, 2023). The assembly was checked for contamination and corrected as described previously ( Howe *et al.*, 2021). Manual curation was performed using gHiGlass ( Kerpedjiev *et al.*, 2018) and PretextView ( Harry, 2022). The mitochondrial genome was assembled using MitoHiFi ( Uliano-Silva *et al.*, 2023), which runs MitoFinder ( Allio *et al.*, 2020) or MITOS ( Bernt *et al.*, 2013) and uses these annotations to select the final mitochondrial contig and to ensure the general quality of the sequence.

A Hi-C map for the final assembly was produced using bwa-mem2 ( Vasimuddin *et al.*, 2019) in the Cooler file format ( Abdennur & Mirny, 2020). To assess the assembly metrics, the *k*-mer completeness and QV consensus quality values were calculated in Merqury ( Rhie *et al.*, 2020). This work was done using Nextflow ( Di Tommaso *et al.*, 2017) DSL2 pipelines “sanger-tol/readmapping” ( Surana *et al.*, 2023a) and “sanger-tol/genomenote” ( Surana *et al.*, 2023b). The genome was analysed within the BlobToolKit environment ( Challis *et al.*, 2020) and BUSCO scores ( Manni *et al.*, 2021; Simão *et al.*, 2015) were calculated.

Table 3 contains a list of relevant software tool versions and sources.

**Table 3.** Software tools: versions and sources.

Software tool	Version	Source
BlobToolKit	4.2.1	https://github.com/blobtoolkit/ blobtoolkit
BUSCO	5.3.2	https://gitlab.com/ezlab/busco
Hifiasm	0.16.1-r375	https://github.com/chhylp123/ hifiasm
HiGlass	1.11.6	https://github.com/higlass/higlass
Merqury	MerquryFK	https://github.com/ thegenemyers/MERQURY.FK
MitoHiFi	3	https://github.com/marcelauliano/ MitoHiFi
PretextView	0.2	https://github.com/wtsi-hpag/ PretextView
purge_dups	1.2.5	https://github.com/dfguan/ purge_dups
sanger-tol/ genomenote	v1.0	https://github.com/sanger-tol/ genomenote
sanger-tol/ readmapping	1.1.0	https://github.com/sanger-tol/ readmapping/tree/1.1.0
YaHS	1.2a.2	https://github.com/c-zhou/yahs

### Wellcome Sanger Institute – Legal and Governance

The materials that have contributed to this genome note have been supplied by a Darwin Tree of Life Partner. The submission of materials by a Darwin Tree of Life Partner is subject to the **‘Darwin Tree of Life Project Sampling Code of Practice’**, which can be found in full on the Darwin Tree of Life website here. By agreeing with and signing up to the Sampling Code of Practice, the Darwin Tree of Life Partner agrees they will meet the legal and ethical requirements and standards set out within this document in respect of all samples acquired for, and supplied to, the Darwin Tree of Life Project. 

Further, the Wellcome Sanger Institute employs a process whereby due diligence is carried out proportionate to the nature of the materials themselves, and the circumstances under which they have been/are to be collected and provided for use. The purpose of this is to address and mitigate any potential legal and/or ethical implications of receipt and use of the materials as part of the research project, and to ensure that in doing so we align with best practice wherever possible. The overarching areas of consideration are:

• Ethical review of provenance and sourcing of the material

• Legality of collection, transfer and use (national and international) 

Each transfer of samples is further undertaken according to a Research Collaboration Agreement or Material Transfer Agreement entered into by the Darwin Tree of Life Partner, Genome Research Limited (operating as the Wellcome Sanger Institute), and in some circumstances other Darwin Tree of Life collaborators.

## Data Availability

European Nucleotide Archive: *Eristalinus aeneus*. Accession number PRJEB62161; https://identifiers.org/ena.embl/PRJEB62161 ( Wellcome Sanger Institute, 2023). The genome sequence is released openly for reuse. The *Eristalinus aeneus* genome sequencing initiative is part of the Darwin Tree of Life (DToL) project. All raw sequence data and the assembly have been deposited in INSDC databases. The genome will be annotated using available RNA-Seq data and presented through the Ensembl pipeline at the European Bioinformatics Institute. Raw data and assembly accession identifiers are reported in Table 1.

## Author information

Members of the Natural History Museum Genome Acquisition Lab are listed here: https://doi.org/10.5281/zenodo.7139035.

Members of the Darwin Tree of Life Barcoding collective are listed here: https://doi.org/10.5281/zenodo.4893703.

Members of the Wellcome Sanger Institute Tree of Life Management, Samples and Laboratory team are listed here: https://doi.org/10.5281/zenodo.10066175.

Members of Wellcome Sanger Institute Scientific Operations: Sequencing Operations are listed here: https://doi.org/10.5281/zenodo.10043364.

Members of the Wellcome Sanger Institute Tree of Life Core Informatics team are listed here: https://doi.org/10.5281/zenodo.10066637.

Members of the Tree of Life Core Informatics collective are listed here: https://doi.org/10.5281/zenodo.5013541.

Members of the Darwin Tree of Life Consortium are listed here: https://doi.org/10.5281/zenodo.4783558.
