# Supplementary material for: The genome sequence of a hoverfly  Eristalinus aeneus (Scopoli, 1763)
Source: Wellcome Open Res. 2024 May 24;9:69. Originally published 2024 Feb 19. [Version 2] doi: 10.12688/wellcomeopenres.20636.2 (PMC11134147; doi:10.12688/wellcomeopenres.20636.2)
Supplement: Peer review history

# Reviewer report: Reviewer response for version 2

**DOI:** 10.21956/wellcomeopenres.24731.r82090

**Published:** 2024-08-08

**Authors:** Andrzej Grzywacz

**Affiliations:** 1 Nicolaus Copernicus University in Toruń, Lwowska, Toruń, Poland

Well written report of *Eristalinus aeneus *genome. Contribution is satisfactory and contains all the necessary information. Introduction covers most important aspects of *Eristalinus aeneus* life history with the most recent references. The genome assembly is of high quality. A brief explanation of taxonomic issues of *Eristalinus aeneus* justify obtaining the genome of this species.

**Only a few minor comments listed below:**

- Should "Aster" be in italics?
- Please merge "A female *Eristalinus aeneus* (specimen ID NHMUK014537016, ToLID idEriAene1) was collected from Mullion, Cornwall, England. The Lizard National Nature Reserve, England, UK (latitude 50.02, longitude –5.24) on 2021-07-01 using an aerial net." into a single sentence.
- "Hi-C data were also generated from remaining tissue of idEriAene1..." please be more specific.

Are sufficient details of methods and materials provided to allow replication by others?

Yes

Is the rationale for creating the dataset(s) clearly described?

Yes

Are the datasets clearly presented in a useable and accessible format?

Yes

Are the protocols appropriate and is the work technically sound?

Yes

Reviewer Expertise:

Entomology, Systematics, Taxonomy

I confirm that I have read this submission and believe that I have an appropriate level of expertise to confirm that it is of an acceptable scientific standard.

# Reviewer report: Reviewer response for version 2

**DOI:** 10.21956/wellcomeopenres.24731.r82085

**Published:** 2024-06-06

**Authors:** Claus Vogl

**Affiliations:** 1 Vetmeduni Vienna, Veterinärplatz, Wien, Austria

The article presents the genome of the hoverfly *Eristalinus aeneus*. The species seems identified cleanly. I am not sure that an insect with such a large distribution range is really one species and not subdivided into many phenotypically similar cryptic species. But only data sets, such as this one will help to answer this question. In any case, the location where the sequenced specimen was collected was given accurately.

 Generally, the authors left little to criticise. On page 3, the following change may clarify the meaning: "In the Afrotropics it extends south to Tanzania and in the Nearctic it reaches California and Texas ()" to "In the Afrotropics it extends south to Tanzania and in the Nearctic south to California and Texas ()".

 And parentheses may help here: "... ragworts and groundsels (Senecio), ..."

 Otherwise the methods seem state of the art, the quality criteria seem to have been met, and the genome appears to be sound. I did not check if I could actually download the genome, but everything seems OK.

Are sufficient details of methods and materials provided to allow replication by others?

Yes

Is the rationale for creating the dataset(s) clearly described?

Yes

Are the datasets clearly presented in a useable and accessible format?

Yes

Are the protocols appropriate and is the work technically sound?

Yes

Reviewer Expertise:

Statistics, population genetics and genomics. I have collaborated on fly genome projects.

I confirm that I have read this submission and believe that I have an appropriate level of expertise to confirm that it is of an acceptable scientific standard.

# Reviewer report: Reviewer response for version 2

**DOI:** 10.21956/wellcomeopenres.24731.r84476

**Published:** 2024-05-28

**Authors:** Ljiljana Šašić Zorić

**Affiliations:** 1 BioSense Institute, University of Novi Sad, Novi Sad, Serbia

Do not have any further comments.

Are sufficient details of methods and materials provided to allow replication by others?

Yes

Is the rationale for creating the dataset(s) clearly described?

Yes

Are the datasets clearly presented in a useable and accessible format?

Yes

Are the protocols appropriate and is the work technically sound?

Yes

Reviewer Expertise:

Population genetics and molecular taxonomy

I confirm that I have read this submission and believe that I have an appropriate level of expertise to confirm that it is of an acceptable scientific standard.

# Reviewer report: Reviewer response for version 1

**DOI:** 10.21956/wellcomeopenres.22839.r80211

**Published:** 2024-05-21

**Authors:** Chufei Tang

**Affiliations:** 1 Jiangsu Academy of Agricultural Sciences, Nanjing, China

This article presents the genome assembly of a cosmopolitan species, *Eristalinus aeneus*, known for its fascinating habits such as potential salt tolerance and pollination. The rationale for creating the dataset is well-explained and highlights its importance. The protocols are suitable and the work is technically sound. Sufficient details of methods and materials are provided to allow replication by others, and the datasets are presented in a clear and accessible format.

 One minor suggestion: the introduction could be better organized - there are eight paragraphs, some of which are quite short. It is suggested to integrate information about adults and larvae together. For example, the study of larvae in the 6th paragraph could be incorporated into the discussion of adult morphology in the first paragraph. Maybe the introduction could be divided as follows.

 1) The species range, as discussed in the first, second, and fourth paragraphs, pertains to the representativeness of the species.

 2) The morphology, including the information in the first and potential additional details from the references cited in the 6th paragraph.

 3) The habits and habitats, maybe first adults then larvae, including the information from the third, fourth and fifth paragraphs.

 4) The progress on the molecular data, including this species and the sister species mentioned in the last paragraph.

 5) The aim of the paper.

Are sufficient details of methods and materials provided to allow replication by others?

Yes

Is the rationale for creating the dataset(s) clearly described?

Yes

Are the datasets clearly presented in a useable and accessible format?

Yes

Are the protocols appropriate and is the work technically sound?

Yes

Reviewer Expertise:

taxonomy, phylogeny

I confirm that I have read this submission and believe that I have an appropriate level of expertise to confirm that it is of an acceptable scientific standard.

# Reviewer report: Reviewer response for version 1

**DOI:** 10.21956/wellcomeopenres.22839.r80217

**Published:** 2024-05-13

**Authors:** Ljiljana Šašić Zorić

**Affiliations:** 1 BioSense Institute, University of Novi Sad, Novi Sad, Serbia

“The genome sequence of a hoverfly, *Eristalinus aeneus* (Scopoli, 1763)” is presented in the form of a technical report on the genome assembly of hoverfly species *Eristalinus aeneus*. The article is an important contribution to further research on hoverflies (Diptera, Syrphidae). It describes technical aspects of genome assembly clearly and concisely. The applied approach includes PacBio and Hi-C data. The methodology is suitable and overall, very well described. I have noticed only few small inconsistencies:

- No comma is needed in the title;
- I am not familiar with the word “nan” which is listed in the keywords.  It is mentioned only at this point in the text. Please check if the spelling is correct;
- Please check the total number of scaffolds. In the text and table 1 it is 197 but based on figures 2 – 4 it should be 198;
- Since abbreviation WSI was introduced for the Wellcome Sanger Institute, please use it consistently in the text.

Are sufficient details of methods and materials provided to allow replication by others?

Yes

Is the rationale for creating the dataset(s) clearly described?

Yes

Are the datasets clearly presented in a useable and accessible format?

Yes

Are the protocols appropriate and is the work technically sound?

Yes

Reviewer Expertise:

Population genetics and molecular taxonomy

I confirm that I have read this submission and believe that I have an appropriate level of expertise to confirm that it is of an acceptable scientific standard, however I have significant reservations, as outlined above.
